# Hyphenation of 2D NMR With Hydrogenative PHIP

**DOI:** 10.1002/mrc.5510

**Published:** 2025-01-22

**Authors:** Bono O. Jimmink, Marco Tessari, Arno P. M. Kentgens

**Affiliations:** ^1^ Institute for Molecules and Materials Radboud University Nijmegen The Netherlands

**Keywords:** ^1^H, homogeneous hydrogenation, hyperpolarisation, isotropic mixing, NMR, Parahydrogen, PASADENA, PHIP, polarisation transfer, TOCSY

## Abstract

Parahydrogen induced polarisation (PHIP) is often used to enhance the sensitivity of NMR, with the purpose of extending the applicability of the technique. Nuclear spin hyperpolarisation obtained via PHIP is generally localised on the protons derived from the addition of para‐enriched hydrogen to an unsaturated substrate. This limitation has been previously addressed by pulse schemes that can spread this hyperpolarised magnetisation through the entire network of J‐coupled protons in the product molecule. Here, we extend this approach, by implementing 2D NMR spectroscopy on such network of hyperpolarised protons. This novel approach to 2D acquisition during parahydrogenation allows information to be gained from the entirety of a molecule, quicker and/or at lower concentrations than by conventional NMR. The efficacy of the method is exemplified by performing a 2D TOCSY experiment during hydrogenative PHIP, using 2‐pentyn‐1‐ol as a substrate. A 2D spectrum was obtained in a few minutes at micromolar concentration, demonstrating the applicability of this methodology.

## Graphical abstract

PHIP provides enhanced magnetisation localised on the protons deriving from para‐enriched hydrogen. By spreading this hyperpolarisation through the *J*‐coupled protons of the hydrogenation product it is possible to acquire a 2D‐PHIP experiment that yields information on the entire spin network at low concentration.

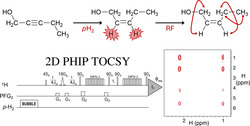

**Abbreviations**
ALTADENA: adiabatic longitudinal transport after dissociation engenders nuclear alignment
PASADENA: parahydrogen and synthesis allow dramatically enhanced nuclear alignment
PHIP: parahydrogen induced polarisation
SABRE: signal amplification by reversible exchange
SEPP: selective excitation of polarisation using PASADENA

## 1 Introduction

Parahydrogen induced polarisation (PHIP) is an umbrella term for several techniques which utilise para‐enriched hydrogen as a source of nuclear spin hyperpolarisation [1, 2] to enhance the sensitivity of NMR. The origin of the NMR signal enhancement in PHIP lays in the singlet order of molecular hydrogen, i.e., the imbalance between the populations of the symmetric (*ortho*) and antisymmetric (*para*) spin configurations relative to thermal equilibrium. In the case of hydrogen molecules, such imbalance can be generated quickly by cooling the gas in the presence of an appropriate ‘spin conversion catalyst’ (commonly activated charcoal doped with iron particles) and allowing the gas to heat back to room temperature in the absence of this catalyst [3, 4, 5, 6]. This process can be performed at relatively low cost and the resulting para‐enriched hydrogen can be stored for weeks. When transferred to substrate molecules by a chemical reaction (e.g., hydrogenative PHIP) [7, 8, 9, 10] or transient association (i.e., SABRE) [2, 11, 12], this singlet order can provide a large increase of nuclear spin polarisation by an appropriate conversion sequence.

The outcome of hydrogenative PHIP experiments strongly depends on whether the chemical reaction is performed at low magnetic field (ALTADENA) [10] or at high magnetic field, inside the magnet of the NMR spectrometer (PASADENA) [8, 9]. Here, we have favoured the latter method for its simpler hardware requirements, as no field cycling is needed for the NMR measurement. In PASADENA experiments, however, only the protons originating from para‐enriched hydrogen exhibit signal enhancement, which significantly limits the information that can be obtained on the structure of the hydrogenation product [1, 8, 9]. Sengstschmid et al. have previously shown that, by combining PASADENA with isotropic mixing in the so‐called SEPP‐TOCSY pulse sequence, this hyperpolarised magnetisation can be spread over a network of J‐coupled protons, thereby regaining important structural information on the product molecule [13]. Here, we expand on this idea, by demonstrating the use of SEPP‐TOCSY as a preparation block for performing hyperpolarised 2D NMR spectroscopy during a parahydrogenation reaction. Previous 2D PHIP‐NMR spectroscopy has mainly aimed at the observation of (intermediate) catalytic species [14, 15], utilising experiments such as 2D‐COSY [16, 17, 18], 2D‐NOESY [16, 17, 18], 2D‐HSQC [16, 17, 18] and 2D‐HMQC [16, 19, 20], on the hyperpolarised protons deriving from para‐enriched hydrogen. Analogously, 2D PHIP‐NMR experiments to characterise the hyperpolarised product– including 2D‐TOCSY [21], 2D‐HMQC [21], 2D DQF‐COSY [22] and 2D COSY [23]—have generally focused on the hyperpolarised protons derived from the parahydrogenation reaction. Instead, when the SEPP‐TOCSY pulse sequence is used as a preparation period, hyperpolarised proton magnetisation is spread over the product molecule, and 2D NMR experiments can be employed to gain detailed structural information at low concentration and in reduced measuring time. A stringent requirement to acquire such 2D PHIP‐NMR spectra is that a steady‐state concentration of hyperpolarised parahydrogenated molecules is produced in the course of the reaction. An alternative was reported by Ratajczyk et al., who used small flip‐angle 1D experiments interleaved with a 2D experiment [22]. The amplitudes of the interferogram could then be adjusted according to the signal amplitude obtained in the corresponding 1D experiments. Here, we demonstrate that, by carefully tuning the experimental conditions, a steady‐state concentration of hyperpolarised reaction product can be obtained. We have tested this novel approach of combining a SEPP‐TOCSY preparation block with 2D NMR, by acquiring a 2D PHIP‐TOCSY of cis‐2‐penten‐1‐ol in 4 min during the parahydrogenation of ca. 75 μM 2‐pentyn‐1‐ol.

## 2 Material and Methods

### 2.1 Chemicals

2‐Pentyn‐1‐ol (CAS:6261‐22‐9), [1,4‐bis(diphenylphosphino)butane](1,5‐cyclooctadiene)rhodium(I) ([Rh (dppb)(COD)]BF4) (CAS:79255‐71‐3), acetonitrile (CAS:75‐05‐8) and methanol‐d_4_ (CAS:811‐98‐3) were purchased from Sigma Aldrich, cis‐2‐penten‐1‐ol (CAS:1576‐95‐0) was purchased from TCI. All chemicals were 97% pure or higher and were used without further purification. A parahydrogen generator (HyperSpin Scientific) cooled by liquid nitrogen at 77 K was used to convert thermal hydrogen (purity 5.0, Linde Gas Benelux B.V.) to 51% para‐enriched H_2_. Nitrogen gas was obtained from the on‐site bulk nitrogen supply (Linde).

^1^H NMR assignment for 2‐penten‐1‐ol: δ 0.983 (t, *J* = 7.6, 3H, C**H**_**3**_{d}), 2.090 (m, *J* = 6.7, 7.5, 1.1, 2H, C**H**_**2**_CH_3_{c}), 4.116 (d, *J* = 5.2, 2H, HOC**H**_**2**_{b}), 5.498 (m, *J* = 1.385, 1.811, 2.131, 4.049, 3.197, 2H, **H**CC**H**{a}). Chemical shifts are given in ppm—referenced to a solvent signal (δ 3.31 methanol‐d_4_ pentet)—and J‐couplings are given in Hz. The letters in curly brackets refer to the signal assignment given in Figure 5.

### 2.2 Solutions for Hydrogenation Reaction

1. Reaction mixtures were prepared from stock solutions of 2‐pentyn‐1‐ol and catalyst precursor [Rh (dppb)(COD)]BF4 in methanol‐d_4_. Hydrogenation reactions were typically performed on a solution of 175 μM 2‐pentyn‐1‐ol in the presence of 75 μM catalyst precursor, and 100 μM acetonitrile. The solution was transferred to a quick pressure valve NMR tube (Wilmad 528‐QPV‐7) and sealed with an in‐house developed headpiece [24]. The sample was cooled in a water bath at 4 °C and purged with nitrogen gas for 10 min. Thereafter, the sample was allowed to heat back up to room temperature before being inserted into the NMR spectrometer. Next, para‐enriched hydrogen gas was bubbled through the solution for approximately 1 min to purge the lines of nitrogen gas, pre‐activate the catalyst and stabilise the reaction before starting the actual 2D PHIP‐TOCSY. During all PHIP measurements, para‐enriched hydrogen gas was bubbled through the solution at the beginning of every scan. This process involves four steps under spectrometer control:pressure in the NMR tube is reduced from 5 to 4 bar via a relief valve connected to the vent line (0.25 s)
2. Para‐enriched H_2_ is bubbled through the solution, restoring the tube pressure to 5 bar (0.35 s)
3. After closing the bubbling valve, pressure is applied above the liquid (0.4 s) to stabilise the pressure, which ensures that no additional gas can escape the hydrogen line.
4. NMR sample is allowed to rest (0.7 s) to dampen the turbulent motions resulting from bubbling.

### 2.3 NMR

All NMR experiments were performed on an Agilent VNMRs spectrometer operating at 500 MHz ^1^H resonance frequency, using a room temperature insert. A series of 1D SEPP‐TOCSYs at mixing times ranging between 0 and 80 ms in steps of 5 ms was acquired on a 20 mM solution of cis‐2‐penten‐1‐ol under thermal conditions. An RF‐field strength of 5.3 kHz was used for the DIPSI‐2 isotropic mixing. For each 1D spectrum 8 transients were recorded. A 50 s recycle delay ensured that each transient was recorded from thermal equilibrium. 1D spectra were processed with ssNake [25], using squared cosine‐bell apodisation, prior to zero filling to 32 k points and polynomial baseline correction. A 2D PHIP‐TOCSY data set consisting of 24(t_1_) × 350(t_2_) complex points was acquired in 4 min, with 4 transients per hypercomplex increment. Spectral widths in acquisition and in the indirect dimension were 5500 Hz and 3500 Hz, respectively. Quadrature detection in the indirect dimension was achieved by P/N‐TPPI. Both DIPSI‐2 mixing periods were applied with a 5.3 kHz RF field for 59.3 ms. In addition to the hydrogen bubbling block above, the reaction takes place during a 0.2 s recycle delay and 0.5 s acquisition time. The 2D data set was processed with NMRPipe [26], using squared cosine‐bell apodisation in both dimensions, prior to zero filling to 1536(t_1_) × 8192(t_2_) complex points, and Fourier transformation. Before FT in the indirect dimension P/N interferograms were combined to obtain cosine‐ and sine‐ amplitude modulation in t_1_.

## 3 Results and Discussion

**FIGURE 1:**
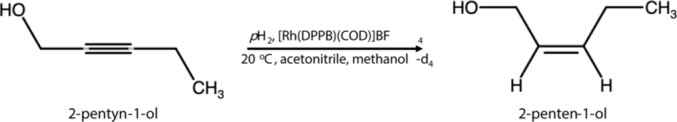
Reaction scheme of the hydrogenation of 2‐pentyn‐1‐ol to 2‐penten‐1‐ol.

Acquisition of a 2D PHIP‐TOCSY spectrum was performed during the parahydrogenation of 2‐pentyn‐1‐ol to cis‐2‐penten‐1‐ol, sketched in Figure 1.

Notably, 2‐penten‐1‐ol is itself unsaturated and can undergo hydrogenation under the applied reaction conditions. However, this reaction does not take place until all 2‐pentyn‐1‐ol has converted to 2‐penten‐1‐ol, which does not occur for the measurements presented here.

**FIGURE 2:**
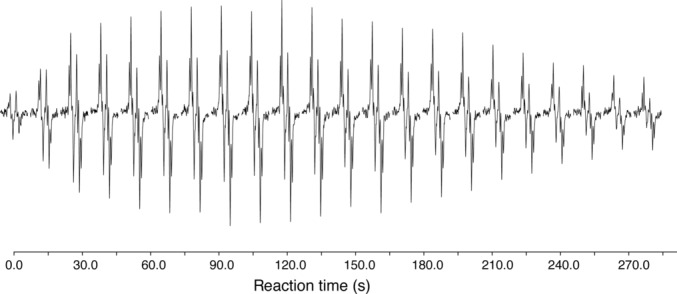
NMR signals of the vinylic protons enhanced via PASADENA during the reaction of 300 μM 2‐pentyn‐1‐ol, 75 μM Rh‐catalyst and 300 μM acetonitrile in methanol‐d_4_ at 293 K.

Acetonitrile, which acts as a competing ligand for the rhodium catalyst, was added to the reaction mixture [27]. This sufficiently slows down the reaction rate for acquiring a complete 2D experiment, while allowing for higher catalyst concentrations, which has a stabilising effect on the reaction profile. As a result, a steady state production of PHIP‐hyperpolarised vinylic proton could be sustained for at least 5 min during the reaction, using 175 μM initial substrate concentration. An illustrative example of a reaction profile is shown in Figure 2, displaying the PASADENA‐enhanced NMR signals of the vinylic protons of 2‐penten‐1‐ol in the course of parahydrogenation.

**FIGURE 3:**
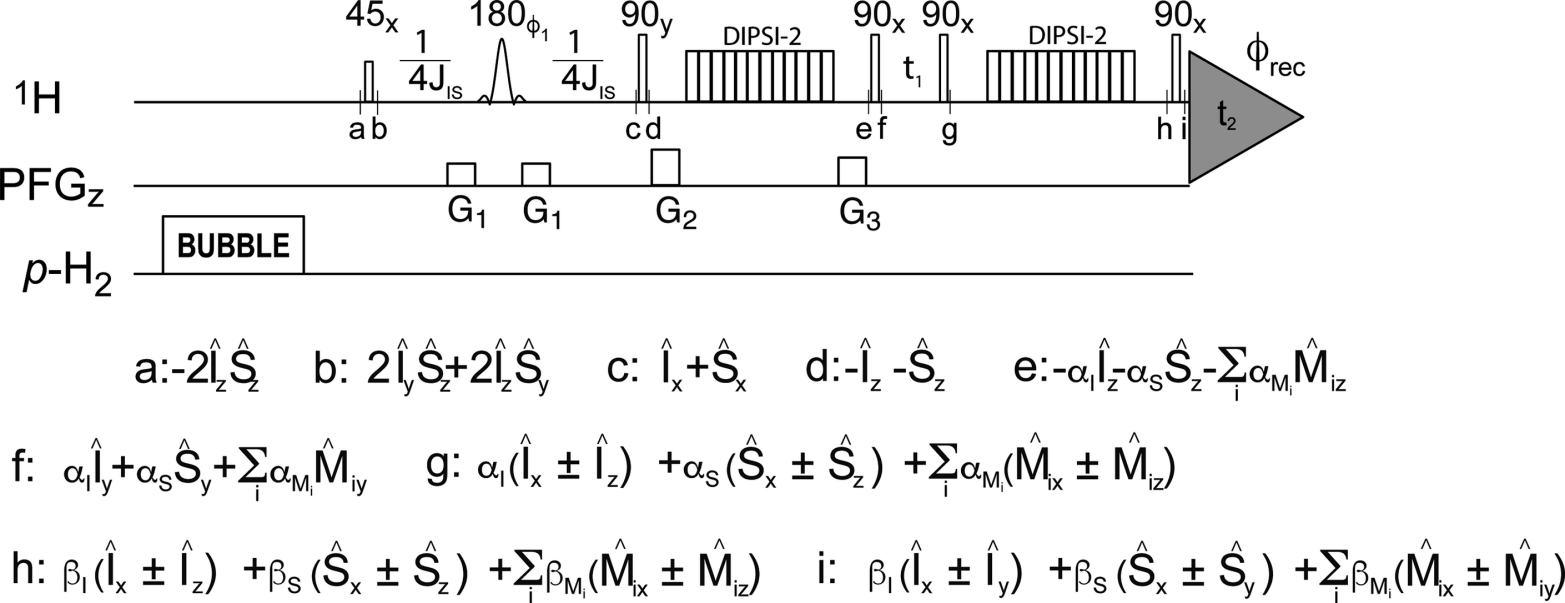
2D PHIP‐TOCSY pulse sequence. The flip angle and phase of each pulse is indicated. The shaped *rSNOB* pulse [28] was selectively applied on the vinylic protons. An RF‐field strength of 5.3 kHz was employed for both DIPSI‐2 isotropic mixing pulse trains, applied in the centre of the aliphatic region (2.2 ppm). Phase cycling: φ_1_ = y, −x, φ_rec_ = x, −x. Pulsed Field Gradients along z were of 1 ms duration and relative amplitudes: G_1_:G_2_:G_3_:G_4_:G_5_ = 1:1.4:1.4:7.6:2.4. J_IS_ indicates the scalar coupling constant between the vinylic protons originating from para‐enriched hydrogen. The duration of both DIPSI‐2 isotropic mixing periods was set to 59.3 ms for optimal magnetisation transfer. Relevant terms of the density operator are reported along the pulse sequence at given time points, where I and S indicate vinylic protons and M_i_ indicates other protons in the J‐coupled network. Note that both cosine and sine modulated terms are retained from the evolution period, allowing for PEP acquisition [29]. Quadrature detection in the indirect dimension was achieved by P/N‐TPPI.

This steady state PHIP hyperpolarisation was employed to acquire a 2D PHIP‐TOCSY spectrum of cis‐2‐penten‐1‐ol, using the pulse sequence shown in Figure 3, with the period between point ‘a’ and ‘f’ serving the purpose of preparing the magnetisation for the subsequent evolution period in a standard 2D acquisition scheme. Bubbling para‐enriched hydrogen at the beginning of each transient maintains a constant H_2_ singlet order throughout the acquisition of the 2D PHIP‐TOCSY experiment. Parahydrogenation of asymmetric molecules at high magnetic field results in longitudinal two‐spin order associated with the vinylic protons [1, 28].

Conversion of this spin order to enhanced magnetisation can be achieved via the SEPP pulse scheme, based on the selective excitation of one of the two protons derived from para‐enriched hydrogen [13].

This approach, however, requires the frequencies of the vinylic protons to be well separated. As shown in Figure 2, the resonances of the vinylic protons are in the present case too close for a selective excitation and, therefore, a π/4 pulse is used to produce antiphase coherence (time point ‘b’). Refocusing to in‐phase magnetisation via scalar coupling occurs after an echo of 1/2 J_IS_ duration (time point ‘c’), which is performed using a selective shaped *rSNOB* refocussing pulse. Note that a two‐step phase‐cycle on the *rSNOB* refocusing pulse [30], allows suppressing thermal magnetisation, thereby selecting only the signals originating from PHIP. Subsequently, the hyperpolarised in‐phase magnetisation is spread via the first DIPSI‐2 isotropic mixing [29, 31], reaching the most distant protons in the J‐coupled network. In Figure 3, the efficiency of the magnetisation transfer from the originally hyperpolarised protons (I and S) to the rest of the J‐coupled network (M_i_), is indicated with a factor α. The duration of the DIPSI‐2 period influences the efficiency of the transfer as well as the maximum ‘distance’—in terms of J‐network—from the protons initially enhanced by PHIP, i.e., it modulates the α factors. During the t_1_ evolution period, proton magnetisation evolves under the effect of offset and scalar coupling interactions (time point ‘f’). Notably, both cosine and sine modulation components are retained during the second isotropic mixing, which allows for the preservation of equivalent pathways (PEP) sensitivity enhancement scheme [32]. The duration of the second DIPSI‐2 isotropic mixing (between time points ‘g’ and ‘h’) determines whether direct or long‐range connectivities along the aliphatic chain are to be observed, which is reflected by the factors β (time point ‘h’). The final 90° pulse excites proton magnetisation for detection (time point ‘i’).

**FIGURE 4:**
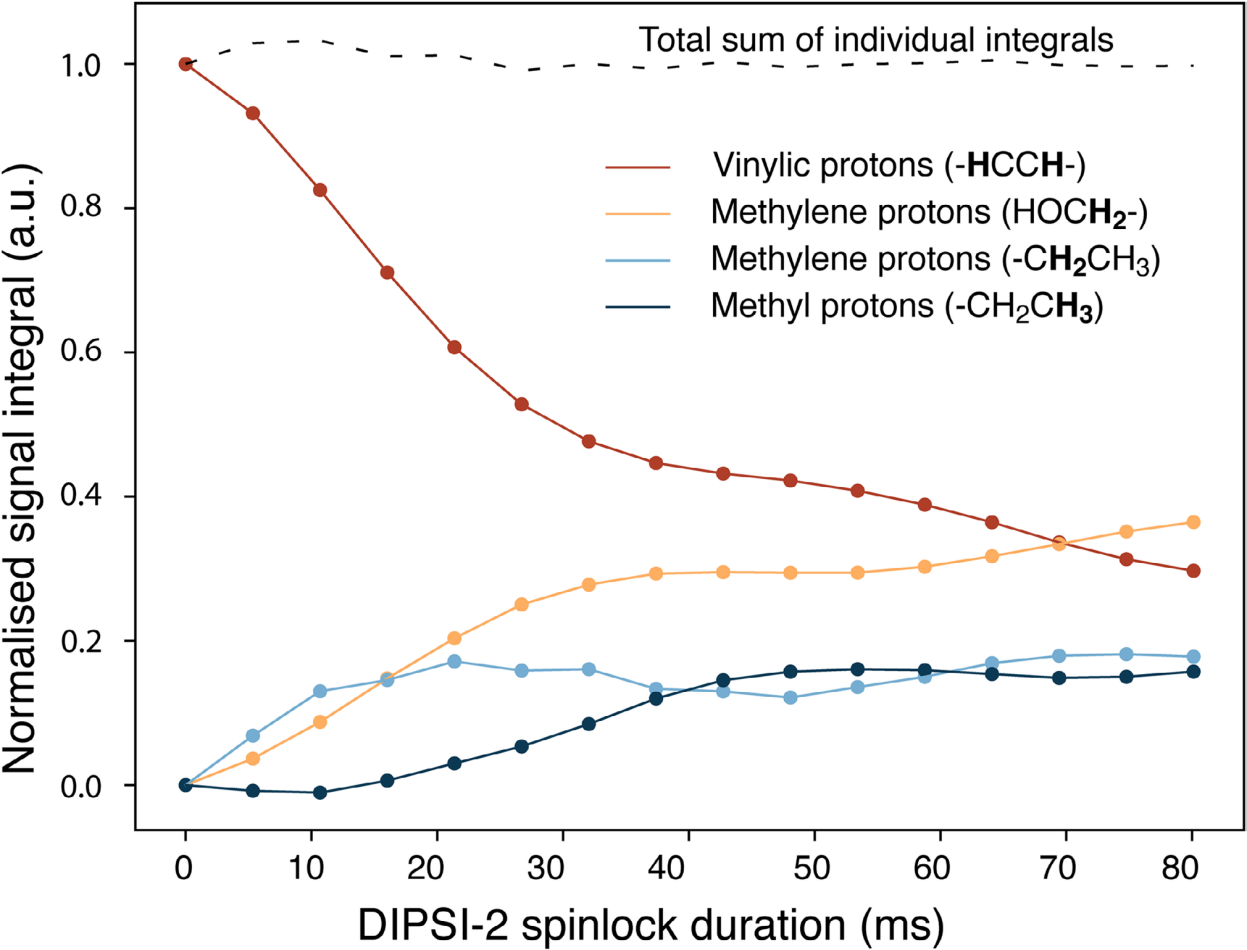
Normalised signal integrals of the four signals of cis‐2‐penten‐1‐ol versus duration of isotropic mixing realised by the DIPSI‐2 pulse‐train (5.3 kHz RF field). The signal originates from the thermal magnetisation of vinylic protons. The dotted line indicates the sum of the signal integrals per time‐point. The NMR sample contained 20 mM cis‐2‐penten‐1‐ol in methanol‐d_4_.

The transfer efficiency of DIPSI‐2 from the vinylic protons through the J‐coupled network was probed with a 20 mM solution of cis‐2‐penten‐1‐ol starting from thermal magnetisation. Figure 4 shows the integrated signal intensity for the four proton signals of cis‐2‐penten‐1‐ol as a function of the DIPSI‐2 mixing time. As expected, magnetisation propagates quickly to the methylene moieties adjacent to the vinylic protons but it takes much longer to reach the methyl group. Note that the sum of the four signal integrals is approximately constant over time indicating a negligible effect of spin relaxation for mixing times up to 80 ms.

**FIGURE 5:**
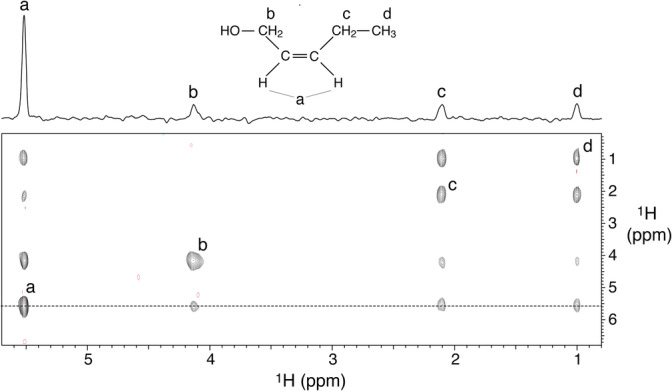
2D PHIP‐TOCSY spectrum acquired at 293 K in 4 min at 500 MHz, ^1^H resonance frequency, during hydrogenation of 175 μM 2‐pentyn‐1‐ol in methanol‐d_4_ using 51% para‐enriched hydrogen, in the presence of 75 μM Rh catalyst and 100 μM acetonitrile. The structure of the reaction product, cis‐2‐penten‐1‐ol, together with the ^1^H signal assignment is shown. The duration of both DIPSI‐2 isotropic mixing periods was set to 59.3 ms at an RF field strength of 5.3 kHz.

The 2D PHIP‐TOCSY spectrum shown in Figure 5 was acquired in 4 min during the parahydrogenation of 2‐pentyn‐1‐ol to cis‐2‐penten‐1‐ol, using the pulse sequence displayed in Figure 3, with isotropic mixing durations of 59.3 ms for both DIPSI‐2 pulse trains. The spectrum displays the expected TOCSY connectivities, except for those between the *c*,*d* protons and the *b* methylene at the other side of the vinylic group, that would require a longer mixing time for the second DIPSI‐2 period to be observable. Conversely, reducing the duration of this mixing to ca. 10 ms would display only the direct connectivities in the product molecule. At the end of the 2D experiment, approximately 75 μM 2‐pentyn‐1‐ol were converted to cis‐2‐penten‐1‐ol by parahydrogenation, which corresponds to a steady state concentration of approximately 1.5 μM hyperpolarised vinylic protons at the beginning of each transient. While the PASADENA‐based enhancement observed on the vinylic protons at higher concentrations is consistent with the published literature (ca. 500‐fold enhancement), an experimental determination of the signal increase in the 2D PHIP‐TOCSY compared to thermal equilibrium is not feasible in the low‐micromolar range.

## 4 Conclusions

PASADENA‐type experiments can be efficiently combined with conventional 2D NMR methods, allowing fast acquisition during a parahydrogenation reaction. By optimising the relative concentrations of homogeneous catalyst, substrate and competing ligands, it is possible to generate a steady state concentration of hyperpolarised molecules, compatibly with the requirements of 2D NMR acquisition. In the present case, a concentration of 1.5 μM cis‐2‐penten‐1‐ol was produced per transient, which proved sufficient to acquire a 2D PHIP‐TOCSY spectrum displaying the expected connectivities. The 2D‐TOCSY experiment was exemplified as it is a versatile experiment for structure elucidation. 2D PHIP can be extended to other homonuclear or heteronuclear NMR experiments, offering a potential tool for the analysis of small unsaturated molecules at low concentration. This approach works well for relatively small J‐coupled networks—e.g., less than 15 distinct resonances of coupled nuclei—so that DIPSI‐2 isotropic mixing can quickly spread the hyperpolarised proton magnetisation through the product molecule. For larger system, the proposed 2D PHIP‐NMR technique is expected to perform less efficiently, due to the excessive dilution of hyperpolarisation among the protons in the J‐network, and the longer duration of the isotropic mixing.

## Author Contributions

**Bono O. Jimmink:** investigation, data acquisition, data processing, writing – original draft preparation. **Marco Tessari:** supervision, data processing, writing – review and editing. **Arno P. M. Kentgens:** supervision, writing – review and editing.

## Data Availability

The data that support the findings of this study are available from the corresponding author upon reasonable request.
